# Phytochemicals of *Euphorbia lathyris* L. and Their Antioxidant Activities

**DOI:** 10.3390/molecules22081335

**Published:** 2017-08-18

**Authors:** Lizhen Zhang, Chu Wang, Qiuxia Meng, Qin Tian, Yu Niu, Wei Niu

**Affiliations:** 1 School of Life Science, Shanxi University, Taiyuan 030006, China; 15835107995@163.com (C.W.); 18834823413@163.com (Q.T.); 2 Institute of Agricultural Environment and Resources, Shanxi Academy of Agricultural Sciences, Taiyuan 030006, China; qiuxia_meng@126.com; 3 Institute of Agricultural Resources and Economic Research, Shanxi Academy of Agricultural Sciences, Taiyuan 030006, China; kayneo@126.com; 4 Shanxi Academy of Agricultural Sciences, Taiyuan 03006, China; niuwillame@163.com

**Keywords:** Caper spurge, phenolic compounds, flavonoids, HPLC, antioxidative activities

## Abstract

The objectives of this study were to characterize the antioxidant capacities and phytochemicals such as phenolics and flavonoids in four parts of *Euphorbia lathyris* L. HPLC was employed to detect the type and content of phenolic acids and flavonoids in the root, stem, seed, and testa of the plant. The total phenolic content (TPC) and total flavonoid content (TFC) were different among various parts of *E. lathyris*. The highest TPC were found in the testa (290.46 ± 15.09 mg of gallic acid equiv/100 g dry weight (DW)). However, the root contained the highest TFC (215.68 ± 3.10 mg of rutin equiv/g DW). Of the different antioxidant activities detected, DPPH free radical scavenging activity was highest in the testa (61.29 ± 0.29 mmol Trolox/100 g DW), but the highest FRAP antioxidant activity was found in the seed (1131.25 ± 58.68 mg FeSO_4_/100 g DW of free compounds and 1927.43 ± 52.13 mg FeSO_4_/100 g DW of bound compounds). There was a positive correlation between the total phenolic contents and DPPH free radical scavenging activity in different parts of *E. lathyris*.

## 1. Introduction

Caper spurge (*Euphorbia lathyris* L.), a well-known herb plant of the family *Euphorbiaceae*, has been widely cultivated as a bioenergy crop or for traditional Asian medicine. *E. lathyris* is widely distributed in all climate types, including Europe, North and South America, Central Asia, East Asia, and North Africa. In Western Europe and the USA, it is used as a source of bio-fuel and biomass because of its high contents of hydrocarbons and fatty acids [1,2]. In traditional Chinese medicine, its seeds are prescribed to treat hydropsy, ascites, coprostasis, amenorrhea, scabies, and snakebite [3], and it has toxicological effects similar to those of croton oil. Previous biological studies have indicated that it displays antitumor effects in curing leucocythemia, esophagus cancer, and skin cancer [4].

To our knowledge, the phytochemical components of the root, seed, and aerial parts of *E. lathyris* have attracted increasing attention in the past few decades, mostly for human health applications. Phytochemicals of *E. lathyris* have shown the presence of varied metabolites, such as diterpene [5,6,7], euphorbetin, aesculetin, daphnetin, β-sitosterol, kaempferol-3-glucuronide [8], vitexicarpin, artemetin, daucosterol, p-hyfroxybenzoic acid [9], flavones, and flavonol glucosides [10]. The diversity of chemical compounds in this herb has been considered to be of biological interest. Phytochemicals may exist in free and bound forms. Bound form materials are conjugated to the cell wall. To our knowledge, most previous studies have reported different profiles of grains. There is only limited literature on the complete profile (free and bound) of the chemical compounds of herbs.

Medicinal plant parts have multiple biological effects including antioxidant activity. In spite of its hydrocarbon-like compounds and medicinal benefits, no data have been previously reported on the antioxidative activity of *E. lathyris*. Previous studies found that the seeds contained fats, proteins, coumarins, and a series of diterpenoids, but the study of phenolic compounds and flavonoids in different parts of *E. lathyris* is still very rare. Therefore, the objectives of the present study were (1) to determine the phytochemical profiles of the total phenolic and flavonoid contents, including both free and bound forms; and (2) to determine the antioxidant activity in different parts of *E. lathyris*. 

## 2. Results and Discussion

### 2.1. Total Phenolic Content (TPC) of Different Parts of E. lathyris 

This study is the first to report the total phenolic content in *E. lathyris*. The free, bound, and total phenolic contents are presented in Table 1. The free phenolic content in testa was highest *(p <* 0.05) (222.12 ± 12.09 mg gallic acid equiv (GAE)/100 g dry weight (DW)), followed by those in stem (161.17 ± 8.64 mg GAE/100 g DW) and seed (95.04 ± 8.90 mg GAE/100 g DW). The highest bound phenolic content was also found (*p* < 0.05) in testa (68.34 ± 3.00 mg GAE/100 g DW), followed by stem (53.97 ± 6.36 mg GAE/100 g DW) and root (52.55 ± 0.44 mg GAE/100 g DW). Similarly, the total phenolic content (*p* < 0.05) in testa was highest (290.46 ± 15.09 mg GAE/100 g DW), followed by those in stem (215.14 ± 15.00 mg GAE/100 g DW) and root (143.00 ± 5.04 mg GAE/100 g DW). There were significant differences in the total phenolic contents among the four parts (*p* < 0.05). The free phenolic content of testa was significantly higher than those in stem, root, and seed (*p* < 0.05). In addition, the free phenolic content of stem was significantly higher than those in root and seed, but no significant differences in the free phenolic content were found between root and seed (*p* > 0.05). The bound phenolic content of testa was significantly higher than those of stem, root, and seed (*p* < 0.05). In addition, no significant differences in the bound phenolic content were seen between stem and root (*p* > 0.05), but their bound phenolic contents were significantly higher than that of seed (*p* < 0.05). Surveswaran et al. [10] reported that the total phenolic levels of *E. lathyris* *was* 1.15g GAE/100 g of dry sample using aqueous solutions of methanol. This method at best extracted only the free or loosely attached and more readily soluble phenolic compounds in the samples, and did not extract phenolic compounds tightly bound to cell wall materials [11]. Moreover, no alkali hydrolysis or further extraction was performed. However, the value reported in their study was significantly higher than those reported in the present study, which were perhaps influenced by the genotype of *E. lathyris*.

In plants, phenolics that provide many health benefits concentrate in the corn or vegetable. We compared the TPC of different parts of *E. lathyris* in this study. It was found that the TPC of the testa was significantly higher than those in the stem, root, and seed. The same results were obtained in many other economically important fruits, including apple, grape berry, and olive, whose peels (or skins) contained higher TPC than other tissues [12]. The TPC ranged from 290.46 to 117.44 mg GAE/100 g DW among different parts of *E. lathyris*. Our results were in accordance with the data reported by Liu [13], who found that among all of the plant extracts investigated, the total phenolics ranged from 0.57 to 280.46 mg GAE/g. Cai et al. [14] found that phenolic compounds were the dominant antioxidant components in the traditional Chinese medicinal herbs associated with anticancer activity. In the present study, high TPC was obtained from *E. lathyris*, indicating that *E. lathyris* is a good source of antioxidants with health benefits.

### 2.2. Total Flavonoid Content (TFC) of Different Parts of E. lathyris 

This study was the first to report the total flavonoid content in *E. lathyris*. The free, bound, and total flavonoid contents are presented in Table 2. The free flavonoid content in root was highest (*p* < 0.05 (144.67 ± 2.89 mg rutin equiv (RE)/100 g DW), followed by those in testa (42.36 ± 0.80 mg RE/100 g DW) and seed (4.23 ± 0.17 mg RE/100 g DW). The highest bound flavonoid content was also recorded (*p* < 0.05) in root (71.01 ± 0.21 mg RE/100 g DW), followed by those in stem (42.28 ± 2.34 mg RE/100 g DW) and seed (7.49 ± 0.22 mg RE/100 g DW). Similarly, the total flavonoid content (*p* < 0.05) in root was highest (215.68 ± 3.1 mg RE/100 g DW), followed by those in stem (45.43 ± 2.63 mg RE/100 g DW) and testa (43.56 ± 0.93 mg RE/100 g DW). There were significant differences in the bound flavonoid content between the four plant parts (*p* < 0.05). The free flavonoid content of root was significantly higher than those of stem, seed, and testa (*p* < 0.05). The total flavonoid content of root was significantly higher than those of stem, seed, and testa (*p* < 0.05).

Flavonoids are regarded as important antioxidants in herbal medicine [15]. We found that the TFC in root of *E. lathyris* was almost 20-fold higher than that in its seed. The TFC ranged from 11.72 mg RE/100 g DW to 215.68 mg RE/100 g DW among different parts of the plant. It is difficult to directly compare our results with others, because the research on flavonoids in different parts of *E. lathyris* remains scarce. However, when compared with the reported flavonoid contents of other plants, such as *Gordon Euryale* seed (1.39 mg RE/g DW), Chinese dwarf cherry seed (2.17 mg RE/g DW), platycodon root (0.95 mg RE/g DW), bitter apricot seed (0.90 mg RE/g DW), or ginkgo seed (0.62 mg RE/g DW) [13], the TFC of *E. lathyris* was considerably higher. The presence of a high flavonoid content is considered beneficial for health, as flavanoids are known to be antioxidative and anti-inflammatory, and exhibit many other health benefits as well [16,17]. *E. lathyris* is also a promising potential source of flavonoid compounds. However, the identification of the flavonoid compounds of *E. lathyris* remains under-investigated, and thus is worthy of further research.

### 2.3. HPLC Analysis of Phenolic Compounds

**Figure 1:**
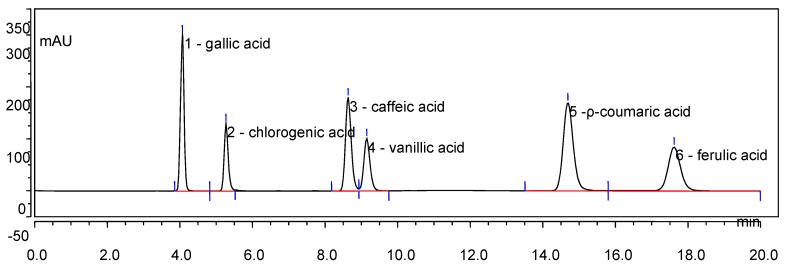
HPLC chromatogram of mixed phenolic acid standards at 280 nm. Gallic acid—4.072 min; chlorogenic acid—5.270 min; caffeic acid—8.635 min; vanillic acid—9.148 min; *p*-coumaric acid—14.692 min; ferulic acid—17.617 min.

**Figure 2:**
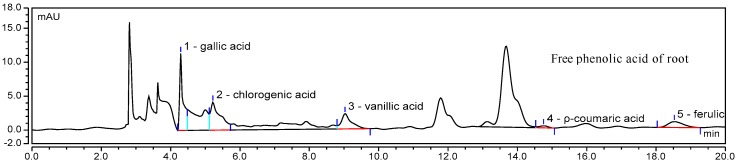
HPLC chromatogram of one sample of phenolic acid.

An HPLC chromatogram of mixed phenolic acid standards at 280 nm is shown in Figure 1, and HPLC chromatograms of one sample are provided in Figure 2. The contents of individual phenolic compounds in different parts of *E. lathyris* are listed in Table 3. 

It was noticed that the free phenolics of the stem included gallic acid, chlorogenic acid, and ferulic acid, while the bound phenolics of stem included gallic acid and chlorogenic acid. Four types of phenolic acids presented in free form in root, namely, gallic acid, chlorogenic acid, vanillic acid, and ferulic acid, while two types of phenolic acids presented in bound form in root, specifically gallic acid and chlorogenic acid. In the seed, gallic acid, vanillic acid, and ferulic acid were found as the three free forms of phenolic acid, while gallic acid and chlorogenic acid presented as two bound form of phenolic acid. 

Caffeic acid and *p*-coumaric acid were only found in the testa. Ferulic acid was significantly higher in testa than in the root, seed, and stem. Chlorogenic acid was significantly higher in root than in the stem, testa, and seed. Jiao et al. [9] detected palmitic acid, benzoic acid, *p*-hydroxybenzoic acid, and oleic acid in the seed of *E. lathyris* on the basis of spectroscopic investigations.

### 2.4. HPLC Analysis of Flavonoids

**Figure 3:**
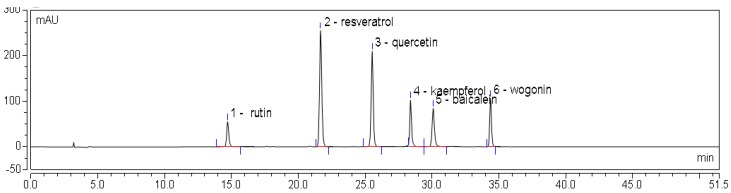
HPLC chromatogram of mixed flavone standards at 360 nm. Rutin—14.743 min; resveratrol—21.642 min; quercetin—25.503 min; kaempferol—28.335 min; baicalein—30.000 min; wogonin—34.287 min.

**Figure 4:**
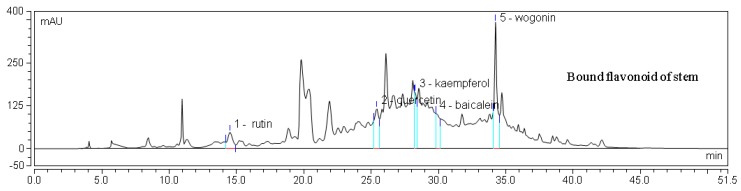
HPLC chromatogram of one sample flavone.

An HPLC chromatogram of mixed flavonoid standards at 280 nm is shown in Figure 3, and HPLC chromatograms for one sample are provided in Figure 4. The contents of individual flavonoids in different parts of *E. lathyris* are listed in Table 4. It was observed that the free flavonoids of the stem included rutin and kaempferol, while the bound flavonoids of the stem included five types of flavonoids, namely, rutin, resveratrol, quercetin, baicalein, and wogonin. In the root, only kaempferol was found as the free form of flavonoid, while quercetin and kaempferol presented as bound flavonoids. Four types of flavonoids presented in free form in seed, namely, rutin, resveratrol, quercetin, and baicalein, while two types of flavonoids presented in bound form in seed, specifically quercetin and kaempferol. Two types of flavonoids presented in free form in testa, namely, rutin and quercetin.

It was found that the levels of rutin, resveratrol, quercetin, baicalein, and wogonin were significantly higher in the stem than those in other parts of the plant. Kaempferol was significantly higher in root than stem. High levels of flavones and flavonol glucosides were identified by HPLC-DAD [10]. In the present study, six flavonoid components were identified in the *E. lathyris* by the HPLC analysis, which were rutin, resveratrol, quercetin, kaempferol, baicalein, and wogonin.

### 2.5. DPPH and FRAP Assays 

The results of DPPH free radical scavenging activity and FRAP values of samples are listed in Table 5. The results showed that there were significant differences in the DPPH radical scavenging activity among the four parts (*p* < 0.05). The highest DPPH free radical scavenging activity was found in extracts of testa in *E. lathyris*, followed by root, seed, and stem. Furthermore, a statistically significant difference in FRAP activity was found among the four parts (*p* < 0.05), with the highest FRAP antioxidant activity found in root, and followed by seed, stem, and testa (*p* < 0.05). 

There was a positive correlation between the total phenolic content and the DPPH free radical scavenging activity in different parts of *E. lathyris* (R^2^ = 0.69, *p* < 0.05). The plant parts containing high total phenolic contents showed higher DPPH free radical scavenging activity. However, the total flavonoid content did not correlate with the DPPH free radical scavenging activity of *E. lathyris* in this study. Both the total phenolic content and total flavonoid content did not correlate with the FRAP antioxidative activity. This could be because the total expressed antioxidative activity may be associated with the relative proportions of each compound in the sample.

A study by Cai et al. reported that medicinal herbs demonstrate much stronger antioxidant activity and contain significantly more phenolics than common vegetables and fruits which are considered as good natural sources of dietary antioxidants [14]. The results of the present study indicate that the extract of *E. lathyris* has significant antioxidant activities, perhaps due to its high contents of phenolics and flavonoids. Many reports have suggested that the antioxidant properties in *Euphorbiaceae* members are mainly due to the presence of high contents of secondary metabolites, such as different types of flavonoids [18,19].

In the present study, twelve standard samples were used in the HPLC analysis, and were also identified in the *E. lathyris* sample, which were gallic acid, chlorogenic acid, vanillic acid, *p*-coumaric acid, ferulic acid, caffeic acid, rutin, resveratrol, quercetin, kaempferol, baicalein, and wogonin. These compounds possess strong antioxidative, antibacterial, and antimutagenic properties. Our results were in accordance with the results of Ho et al. [20], who found that some phenolic acids and flavonoids possessed potent antioxidative activity, in addition to anticancer, anticarcinogenic, and antimutagenic activities. Also, ferulic acid is found in many Chinese medicinal herbs and is an effective antioxidant and vasodilatory agent. However, it is believed that thousands of phenolic compounds occur in medicinal herbs. For instance, more than 4000 kinds of flavonoids and hundreds of coumarins and lignans have been reported as naturally occurring compounds [21]. Therefore, further chemical investigation in the tested herb will be required to reveal various types of phenolic compounds.

Flavonoids and phenolics, which are known as hydrophilic antioxidants, are botanic secondary metabolites that are most abundant in plants [22]. It should be noted that there were several unknown compounds present that were not identified in this study; therefore, more research in this area is warranted.

## 3. Materials and Methods 

### 3.1. Plant Material

*E. lathyris* was planted in Dongyang Traditional Chinese Medicine and Wild Plant Nursery of Shanxi Academy of Agricultural Sciences. Four parts of *E. lathyris* (namely, the stem, root, testa and seed) were collected from 10 plants in October 2012. The samples were separated, weighed, dried in the oven, pulverized and sieved (60 mesh), and finally stored at −20 °C. The plant was identified and authenticated by Dr. Qiuxia Meng of the Institute of Agricultural Environment and Resources, Shanxi Academy of Agricultural Sciences. A voucher specimen (#0201) was deposited at the same institute. 

### 3.2. Chemicals

Gallic acid, chlorogenic acid, vanillic acid, *p*-coumaric acid, ferulic acid, 2,2-diphenyl-1-picrylhydrazyl (DPPH) free radical, sodium borohydride, chloranil, tetrahydrofuran (THF), vanillin, 2,4,6-Tris (2-pyridyl)-s-triazine (TPTZ), and FeSO_4_ were purchased from Sigma Aldrich Co. (St. Louis, MO, USA). Rutin, resveratrol, quercetin, kaempferol, baicalein, and wogonin were purchased from Beijing Century Aoke Biological Technology Co. (Beijing, China). Folin Ciocalteu’s reagent was purchased from Beijing Soledad Bao Technology Co. (Beijing, China). All the other chemicals were of analytical grade or HPLC grade.

### 3.3. Extraction of Samples

Free and bound compounds of samples were extracted using the method reported by Adom et al. [23]. Briefly, a 20-g sample was mixed with 200 mL 80% cold acetone for 10 min in a high-speed homogenizer. The mixture was centrifuged at 2500 rpm for 10 min and the supernatant was collected. The process was repeated three times. The supernatants were pooled and evaporated to dryness at 45 °C with a rotary evaporator to yield the extract. The free extracts were stored at −20 °C until analysis. The residue of the free extraction was digested for 1 h at room temperature in a 1000 mL beaker with 200 mL 2 M NaOH. The mixture was then neutralized with concentrated hydrochloric acid before being extracted with 200 mL ethyl acetate for 10 min. The mixture was then centrifuged at 2500 rpm for 10 min and the supernatant was collected. The process was repeated three times. The supernatants were pooled and evaporated to dryness at 45 °C with a rotary evaporator. The bound extracts were stored at −20 °C until analysis. Prior to analysis, the extracts were resuspended in 10 mL deionized water, respectively.

### 3.4. Determination of Total Phenolic Content (TPC)

The Folin-Ciocalteu colorimetric method was used for total phenolic content of each sample [24]. Briefly, 100 μL resuspended extract (or gallic acid standard solution) was diluted with 400 μL deionized water in a glass culture tube, and 100 μL Folin-Ciocalteu reagent was then added. The mixture was mixed well and incubated at room temperature for 8 min before adding 1 mL 7% sodium carbonate and 0.8 mL deionized water. The mixture was blended well and incubated for 90 min at room temperature. Duplicates of the sample or standard (200 μL each in volume) were transferred to a 96-well plate. Absorbances were measured at 760 nm in a microplate reader. Total phenolic acid content was expressed as milligrams of gallic acid equivalents per 100 g of sample on a dry weight basis (mg GAE/100 g DW).

### 3.5. Determination of Total Flavonoid Content (TFC)

The total flavonoid content of each sample was determined using the sodium borohyride/chloranil-based assay [25]. Tested extracts were added to test tubes and reconstituted in 1 mL of THF/EtOH (1:1, *v*/*v*). Volumes of 0.5 mL 74.6 mM AlCl_3_ and 0.5 mL 50.0 mM NaBH_4_ solution were added to each test tube with 1 mL rutin standard solution or 1 mL sample solution. The tubes were shaken on the Lab-Line orbital shaker for 30 min at room temperature. An additional 0.5 mL 50.0 mM NaBH_4_ solution was added into each test tube with continued shaking for 30 min at room temperature. Then, 2.0 mL cold (4 °C) 0.8 M acetic acid solution was added to each test tube. The solutions were protected from the light and shaken on the orbital shaker at room temperature for 15 min after a thorough mix. Subsequently, 1 mL 20.0 mM chloranil was added to each tube. The tubes were placed in water bath set at 95 °C. The reaction solutions were cooled using tap water. The solutions were transferred to glass culture tubes and brought to a volume of 4.0 mL with methanol. Then, 1 mL 16% vanillin methanol solution was added to each tube and mixed. Then, 2 mL 12 M HCl was added to each tube, mixed, and kept in the dark at room temperature for 15 min. The solutions in the glass culture tubes were centrifuged at 2500 rpm for 3 min. Each solution (200 μL) was added into a 96-well plate in duplicate and absorbances were measured at 490 nm using a microplate reader. Total flavonoid content was expressed as milligrams of rutin equivalents per 100g of sample on a dry weight basis (mg RE/100 g DW).

### 3.6. HPLC Analysis of Phenolic Compounds

Phenolic compounds were separated and identified using an Ultimate3000 C18 column (4.6 mm × 150 mm, 5 µm) (Thermo Electron Corporation, Waltham, MA, USA). The mobile phase was 20% (*v*/*v*) aqueous acetonitrile (pH = 3.0). The volume of injection for both samples and standards was 10 μL. The flow rate was 0.5 mL/min, and the column temperature was 30 °C. Each injection was monitored at 280 nm. Identification of each peak was confirmed using the retention time and absorbance spectrum of each pure compound. Percent recoveries were determined by spiking a known amount of pure compound into a sample and performing the same extraction and analytical procedures. The percent recovery for gallic acid, chlorogenic acid, vanillic acid, *p*-coumaric acid, resveratrol, and ferulic acid were higher than 90% (*n* = 3). The linear relationship of the six phenolic acids and the results of the recovery tests are shown in Table 6.

### 3.7. HPLC Analysis of Flavonoids

The determination of the flavonoid composition was conducted using an HPLC method previously reported [26], employing an Ultimate3000 C18 column (4.6 mm × 150 mm, 5 µm). The mobile phase was a mixture of solvent A (10% (*v*/*v*) aqueous acetonitrile plus 2 mL/L acetic acid) and solvent B (40% acetonitrile, 40% methanol, and 20% water plus 2 mL/L acetic acid). The following gradient (expressed as %B) was used: 0–17 min, 10–42.5%; 17–23 min, 42.5–70%; 23–26.5 min, 70%; 26.5–31.5 min, 70–100%; 31.5–36.5 min, 100%; 36.5–52 min, 10%. The flow rate was 0.5 mL/min, and the column temperature was 30 °C. The volume of injection for both samples and standards was 10 μL. Each injection was monitored at 360 nm. Identification of each peak was confirmed using the retention time and absorbance spectrum of each pure compound. Percent recoveries were determined by spiking a known amount of pure compound into a sample and performing the same extraction and analytical procedures. The percent recovery for rutin, quercetin, kaempferol, baicalein, and wogonin were higher than 90% (*n* = 3). The linear relationship of the six flavones and the results of the recovery tests are given in Table 7.

### 3.8. Ferric-Reducing Antioxidant Power (FRAP) Assay

The FRAP assay was carried out according to the method of Benzie and Strain [27]. Briefly, 100 μL of extracts and 4.9 mL of FRAP reagent, consisting of 100 mM acetate buffer (pH 3.6), 10 mM 4,6-tripryridyls-triazine (TPTZ) in 40 mM HCl, and 20 mM ferric chloride in a 10:1:1 ratio (by volume), were transferred into vials and incubated at 37 °C for 10 min. The FRAP reagent was prepared fresh daily and was warmed to 37 °C in a water bath prior to use. Then, the absorbance at 593 nm was measured relative to a reagent blank also incubated at 37 °C. The FRAP data for samples were determined against the standard of a known FRAP value, FeSO_4_. All solutions were used on the day of preparation. Results were expressed as mg FeSO_4_/100 g DW.

### 3.9. DPPH Free Radical Scavenging Activity

DPPH free radical scavenging activity was measured using a modification of the method by Brand-Williams et al. [28]. Briefly, 2 mL of different concentrations of sample extracts were allowed to react with 4 mL DPPH solution for 30 min in the dark after mixing. The absorbance at 517 nm was measured relative to a reagent blank also incubated under the same conditions. The percentage inhibition was calculated against a control and compared to a Trolox standard curve (60–200 μg/mL). The DPPH radical scavenging rate (%) was calculated as follows:

$$\mathrm{Radical}\;\mathrm{scavenging}\;\mathrm{rate}\;\left(\%\right)\;=\left(1-\frac{A_{x}\times A_{t}\;}{A_{0}}\right)\times 100$$

where *Ax* is the absorbance of test compounds, *A_t_* is the absorbance of test compounds with 4 mL anhydrous ethanol as the blank reaction, and *A*_0_ is the absorbance of ethanol solution (95%, *v*/*v*) as the blank reaction.

The absorbance at 517 nm was measured relative to a reagent blank. The percentage inhibition was calculated against a control and compared to a Trolox standard curve (60–200 μg/mL). 

### 3.10. Statistical Analysis

All extractions and analyses were performed in triplicate. All values were expressed as means ± standard deviation (SD). One-way analysis of variance (ANOVA) followed by Tukey test was computed to determine significant differences between the means by SPSS Statistics 19. A significant difference was defined at *p* < 0.05 or 0.01. Correlation coefficients were determined by the Excel program.

## 4. Conclusions

The results showed that *E. lathyris* is rich in active components, such as phenolics and flavonoids. The highest total phenolic content was recorded in the testa of *E. lathyris*, while the highest total flavonoid content was obtained in the root. Meanwhile, the testa extract of *E. lathyris* showed higher DPPH free radical scavenging activity than root, stem, and seed extracts, but the seed extract showed higher FRAP antioxidant activity. Total phenolic contents in different parts of *E. lathyris* and their DPPH free radical scavenging activity were positively correlated. As the phytochemical contents in different parts of *E. lathyris* varied significantly, we could select appropriate parts to obtain a high yield of target phytochemicals.

To our knowledge, this is the first time that the flavonoids and other phenolics of *E. lathyris* have been analyzed with HPLC, although diterpenoids of *E. lathyris* seed have been measured in the literature without including the phenolic and flavonoid contents in different parts of *E. lathyris.* The analysis of the type and content of polyphenols in different parts of *E. lathyris* can shed a light on the further development and utilization of this Chinese medicinal plant.

## Figures and Tables

**Table 1:** Contents of phenolic acids of *Euphorbia lathyris* (mg galic acid equiv/100 g dry weight (DW)).

Part	Free Phenolics	Bound Phenolics	Total Phenolics
stem	161.17 ± 8.64 ^b^	53.97 ± 6.36 ^b^	215.14 ± 15.00 ^b^
root	90.45 ± 4.60 ^c^	52.55 ± 0.44 ^b^	143.00 ± 5.04 ^c^
seed	95.04 ± 8.90 ^c^	22.40 ± 0.53 ^c^	117.44 ± 9.43 ^d^
testa	222.12 ± 12.09 ^a^	68.34 ± 3.00 ^a^	290.46 ± 15.09 ^a^

Values with different letters in common columns are significantly different (*p* < 0.05, *n* = 3).

**Table 2:** Contents of flavonoids of *Euphorbia lathyris* (mg of rutin equiv/100 g DW).

Part	Free Flavonoids	Bound Flavonoids	Total Flavonoids
stem	3.15 ± 0.29 ^c^	42.28 ± 2.34 ^b^	45.43 ± 2.63 ^b^
root	144.67 ± 2.89 ^a^	71.01 ± 0.21 ^a^	215.68 ± 3.1 ^a^
seed	4.23 ± 0.17 ^c^	7.49 ± 0.22 ^c^	11.72 ± 0.39 ^c^
testa	42.36 ± 0.80 ^b^	1.20 ± 0.13 ^d^	43.56 ± 0.93 ^b^

Values with different letters in common columns are significantly different (*p* < 0.05, *n* = 3).

**Table 3:** Composition and contents of phenolic acid in *Euphorbia lathyris* L. samples (mg/100 g DW).

Part	Composition	Free	Bound	Total
stem	gallic acid	1.16 ± 0.01	0.83 ± 0.04	1.99 ± 0.20
	chlorogenic acid	0.38 ± 0.01	3.53 ± 0.06	3.91 ± 0.07
	vanillic acid	nd	nd	nd
	ferulic acid	0.52 ± 0.10	nd	0.52 ± 0.10
root	gallic acid	1.19 ± 0.15	1.04 ± 0.14	2.23 ± 0.29
	chlorogenic acid	1.03 ± 0.04	7.83 ± 0.15	8.86 ± 0.19
	vanillic acid	0.50 ± 0.06	nd	0.50 ± 0.06
	*p*-coumaric acid	nd	nd	nd
	ferulic acid	0. 97 ± 0.07	nd	0.97 ± 0.07
seed	gallic acid	1.57 ± 0.06	0.95 ± 0.07	2.52 ± 0.13
	chlorogenic acid	nd	0.82 ± 0.03	0.82 ± 0.03
	vanillic acid	0.08 ± 0.01	nd	0.08 ± 0.01
	*p*-coumaric acid	nd	nd	nd
	ferulic acid	0.59 ± 0.06	nd	0.59 ± 0.06
testa	gallic acid	1.79 ± 0.01	nd	1.79 ± 0.01
	chlorogenic acid	2.97 ± 0.18	nd	2.97 ± 0.18
	caffeic acid	5.36 ± 0.21	nd	5.36 ± 0.21
	*p*-coumaric acid	4.88 ± 0.09	nd	4.88 ± 0.09
	ferulic acid	1.18 ± 0.11	nd	1.18 ± 0.11

nd = not detected. Data are expressed as means ± standard deviation (SD) of triplicate samples.

**Table 4:** Composition and contents of flavonoids in the *Euphorbia lathyris* samples (mg/g DW).

	Stem			Root			Seed			Testa		
	Free	Bound	Total	Free	Bound	Total	Free	Bound	Total	Free	Bound	Total
rutin	50.65 ± 0.11	184.37 ± 0.54	235.02 ± 0.65	nd	nd	nd	78.75 ± 0.59	nd	78.75 ± 0.59	107.43 ± 0.18	nd	107.43 ± 0.18
resveratrol	nd	258.17 ± 2.04	258.17 ± 2.04	nd	nd	nd	5.20 ± 0.13	nd	5.20 ± 0.13	nd	nd	nd
quercetin	nd	143.15 ± 1.24	143.15 ± 1.24	nd	78.00 ± 1.56	78.00 ± 1.56	7.47 ± 0.44	16.32 ± 0.39	23.79 ± 0.83	8.73 ± 0.06	nd	8.73 ± 0.06
kaempferol	12.87 ± 0.59	nd	12.87 ± 0.59	9.27 ± 0.86	8.97 ± 0.21	18.24 ± 1.07	nd	6.27 ± 0.25	6.27 ± 0.25	nd	nd	nd
baicalein	nd	119.95 ± 0.36	119.95 ± 0.36	nd	nd	nd	14.47 ± 0.48	nd	14.47 ± 0.48	nd	nd	nd
wogonin	nd	589.40 ± 0.55	589.40 ± 0.55	nd	nd	nd	nd	nd	nd	nd	nd	nd

nd = not detected. Data are expressed as means ± SD of triplicate samples.

**Table 5:** DPPH and FRAP assays of *Euphorbia lathyris*.

	Stem			Root			Seed			Testa		
	Free	Bound	Total	Free	Bound	Total	Free	Bound	Total	Free	Bound	Total
DPPH assay	1.57 ± 0.05e	0.63 ± 0.06f	2.20 ± 0.11d	2.46 ± 0.12c	4.02 ± 0.10b	6.48 ± 0.23a	0.61 ± 0.05f	2.04 ± 0.10d	2.65 ± 0.15c	61.29 ± 0.29a	nd	61.29 ± 0.29a
FRAP assay	105.71 ± 1.51h	71.39 ± 2.95i	177.10 ± 4.46f	1807.50 ± 17.09d	37.69 ± 4.69j	1845.19 ± 21.78c	1131.25 ± 6.38e	1972.43 ± 52.13b	3106.68 ± 58.51a	69.29 ± 4.92i	44.86 ± 0.13j	114.15 ± 5.05g

DPPH free radical scavenging activity values are expressed as mmol Trolox/100 g DW. FRAP activity values are expressed as mg FeSO_4_/100 g DW. Values with different letters for each parameter in the same row are significantly different (*p* < 0.05).

**Table 6:** The linear relationship of the six phenolic acids and the results of recovery tests.

Phenolic Acid	Regression Equation	R^2^	Recovery Rate/%	Relative Standard Deviation (RSD)/%
gallic acid	*Y* = 0.671*X* − 0.4473	0.9995	97.96	0.67
chlorogenic acid	*Y* = 0.3466*X* + 0.7087	0.9998	95.95	0.96
caffeic acid	*Y* = 0.6887*X* + 0.9467	0.9996	99.50	0.30
vanillic acid	*Y* = 0.4037*X* + 0.5282	0.9997	93.87	0.32
*p*-coumaric acid	*Y* = 1.0566*X* + 0.6752	1.0000	97.42	0.15
ferulic acid	*Y* = 0.6427*X* − 0.2327	0.9999	97.34	0.96

**Table 7:** The linear relationship of the six flavones and the results of recovery tests.

Flavone	Regression Equation	R^2^	Recovery Rate/%	RSD/%
rutin	*Y* = 0.2751*X* + 0.7493	0.9994	107.05	0.44
resveratrol	*Y* = 1.3482*X* + 2.1331	0.9993	91.21	5.15
quercetin	*Y* = 0.4254*X* − 0.5830	0.9998	97.68	2.05
kaempferol	*Y* = 0.4856*X* − 1.0943	0.9997	116.54	1.01
baicalein	*Y* = 0.7495*X* − 4.5946	0.9995	95.43	1.74
wogonin	*Y* = 0.3569*X* − 0.4124	0.9996	91.62	1.9
